# In Vitro Antimicrobial, Antioxidant and Anticancer Activities of Egyptian Citrus Beebread

**DOI:** 10.3390/molecules26092433

**Published:** 2021-04-22

**Authors:** Nesren Elsayed, Hatem Sharaf El-Din, Ammar B. Altemimi, Hanaa Y. Ahmed, Anubhav Pratap-Singh, Tarek Gamal Abedelmaksoud

**Affiliations:** 1 Food Science Department, Faculty of Agriculture, Cairo University, Giza 12613, Egypt; tareekgamal_88@agr.cu.edu.eg; 2 Economic Entomology and Pesticides Department, Faculty of Agriculture, Cairo University, Giza 12613, Egypt; sharaf85-0000@agr.cu.edu.eg; 3 Department of Food Science, College of Agriculture, University of Al-Basrah, Basrah 61004, Iraq; ammar.ramddan@uobasrah.edu.iq; 4 The Regional Center for Mycology and Biotechnology, Al-Azhar University, Cairo 11651, Egypt; hanaa_hyk@yahoo.com; 5 Food, Nutrition & Health Program, Faculty of Land and Food Systems, The University of British Columbia, Vancouver, BC V6T 1Z4, Canada

**Keywords:** honeybee, minerals, free sugar, fatty acid, bioactive compounds

## Abstract

In this study, Egyptian beebread (EBB) was investigated for its nutritive value, chemical composition, antioxidant properties, antimicrobial and antitumor activities. Results indicated that EBB was a good source of protein (23.58 ± 0.183 g/100 g BB), total free sugar (20.266 ± 0.930) and potassium (290.202 ± 2.645 mg/100 g). Additionally, 14 fatty acids were identified in EBB, wherein polyunsaturated and monounsaturated fatty acids represented 51.06% ± 0.09% and 9.86% ± 0.01%, respectively. The EBB extract exhibited almost 400% better antiradical activity than BHT, with IC_50_ of EBB extract being 10.7 µg/mL compared to 39.5 µg/mL for BHT. EBB exhibited higher inhibitory activity than the reference compound against *Staphylococcus aureus* and *Escherichia coli*, followed by *Bacillus* subtilis. No inhibitory activity was observed against *Aspergillus Niger*. Additionally, the highest inhibitory activity was recorded against Caco-2 cells, followed by PC3 and HepG-2 cancer cell lines with IC_50_ values 262, 314 and 386 μg/mL, respectively. These findings establish the potential of EBB as an antioxidant, antimicrobial and antitumor agent, with possible applications as natural food supplements and natural preservatives.

## 1. Introduction

There is a growing concern around the utilization of artificial additives in the processed foods such as flavors, coloring agents, emulsifiers, stabilizers and antioxidants. As these chemicals are considered unhealthy and even carcinogenic, consumer demand has driven the exploration for natural antimicrobials in food preservation [1]. Beebread (BB) is one of the valuable honeybee products, which is a mixture of plant pollen and honey fermented with lactic acid and used by bees as a nutritious source of food for both old larvae and workers to stimulate royal jelly production [2]. Beebread is mainly a product of the beehive, as bees firstly collect pollen from flowers on their hind leg and transfer it to the hive, and then workers mix the pollen with their digestive enzymes and honey, which is then followed by lactic fermentation of the mixture to end up with the “Beebread”. Although the production process seems easy and natural, many beekeepers are unable to produce beebread due to the combs damage that happens during the harvesting [2]. Beebread is rich in protein, fat, minerals and vitamins source, in addition to being a source for antioxidant compounds such as α-tocopherol, phenolic compounds and coenzyme Q10 [3]. The chemical composition of beebread varies from one region to another and is dependent on three factors, the surrounding plants, the climatic factors, and the seasonal fluctuations [4].

Studies on beebread are scarce and limited, although a rich body of literature is available for other bee products [5]. To the best of our knowledge, no studies have focused on the full-spectrum characterization of the Egyptian variety of citrus beebread. Beebread has the potential to tackle the problem of multidrug resistance arising due to the overuse of antibiotics [6]. The World Health Organization (WHO) recognized it as an ongoing public health problem [7]. This study explores beebread as an alternative substance, effective against resistant pathogenic microorganisms, which would limit the use of conventional antibiotics [8]. Further, cancer represents one of the five leading causes of death and the most significant cause of death worldwide. We also explore the utilization of beebread phytochemicals as anticancer agents. Increasing evidence suggests beebread’s potential therapeutic benefits, including antioxidant, antitumor [9] and antimicrobial activities [10].

## 2. Results and Discussion

### 2.1. Palynological Examination of the Beebread

The results of pollen analyses showed that beebread samples contained *Citrus* spp. pollen at a range of 90–95% (*w*/*w*). This suggests that beebread characteristics depend on the citrus pollen and nectar nutritional features. Beebread characteristics are expected to be in the same trend as Kaplan et al. [11] who analyzed 8 beebread samples including samples of citrus beebread.

### 2.2. Nutritional Value of Beebread

The chemical analysis in Table 1 showed that carbohydrates and proteins were the main macronutrients in the nutritional composition of beebread (64.10 ± 0.391 and 23.58 ± 0.183 g/100 g BB, respectively) while lipids and ash (9.45 ± 0.079 and 2.87 ± 0.108 g/100 g, respectively) were present in minor concentration. The overall energy (or calorific value) was beebread was found to be 435.77 ± 0.719 kcal/100 g BB. These results agree with Bakour et al. [12] who found the same trend in their research and demonstrated that beebread is a healthy source of protein and carbohydrates. Additionally, Campos et al. [13] showed that carbohydrates comprised close to 55% of the pollen, which is close to our results. As for beebread, bees produce carbohydrate-rich honey during the manufacturing process, so it is expected that the carbohydrates content to be higher in the pollen.

The protein content ranged from 27.6% to 29.9%, respectively in beebread samples, which have been collected from Serbia and Colombia [3]. Additionally, Kaplan et al. [11] showed that the lipid value in beebread samples of different origins ranged from 5.93 to 11.55 g per 100 g beebread. The amount of lipid can also be affected by the plant origin due to the percent of unsaturated fatty acids in the pollen composition [14]. While Zuluaga et al. [3] reported that the amount of lipid ranged from 1.65% to 5.50%, the lipid content generally fluctuates between 4.51% and 4.92% in Serbian beebread [15].

Moreover, the quantity of ash (that reveals the mineral content) agrees with the literature for bee pollen, as it should not exceed 6% [13]. The energy value resulting from consuming beebread is assessed to be on average 435.77 kcal/100 g. This value was lower than the values reported by Bakour et al. [12] for multifloral beebread obtained from Morocco, which refers to the differences in carbohydrate ratio. Additionally, the variations found in the chemical composition of beebread could relate to the floral source and seasonal variations in the pollen and the accessibility of various species of plants [2]. So, all these results confirmed that beebread is a rich nutritional source.

The determination of free sugar, shown in Table 1, revealed that fructose is the largest constituent (11.355% ± 0.555%) of the beebread followed by glucose (7.136% ± 0.452%) and sucrose (1.775% ± 0.017%). Urcan et al. [2] reported that the beebread also contains small amounts of disaccharides isomaltose, trehalose and turanose. However, there exists a low proportion of sucrose as it primarily comprises monosaccharides decomposed by lactic fermentation. Sugars are considered the core of calorie expenditure for bees, and worker bees need 4 mg of utilizable sugars daily to survive.

#### 2.2.1. Mineral Determination

Mineral determination of beebread (Table 2) showed that the major minerals were potassium (290.202 ± 2.645 mg/100 g of BB), phosphorus (215.202 ± 1.103 mg/100 g of BB) and calcium (170.2 ± 3.995 mg/100 g of BB), while the remaining minerals appeared in lower concentrations (60.1 ± 4.369, 31.9 ± 1.153, 118 11.250 ± 0.687, 8.730 ± 0.644 and 2.66 ± 0.251 mg/100 g of BB for magnesium, iron, sodium, zinc and manganese, respectively). The same trend was observed in a study by Stanciu et al. [16] as they found that the main mineral was potassium, then phosphorus, calcium and finally magnesium, which is consistent with those illustrated in our research.

This finding was also reported by Andjelkovic et al. [15] who compared beebread and the pollen. Their results showed that the content of calcium, potassium, magnesium, iron and phosphorus increased in beebread compared to pollen. Meanwhile, the content of Mn and Zn decreased. This decrease is due to the microbial activity and chemical reactions that occur during beebread production. Several studies suggest variations in mineral content of beebread owing to the nectar of flowers, water, soil and geographical conditions [15].

In addition to that, Loper et al. [17] reported that potassium is the highest mineral in almond beebread, after calcium, magnesium and zinc. Similar results are registered in beebread from the Transylvania region, Romania [16]. The primary source of these minerals is the pollen of plant, geographical conditions, water and nectar composition [15].

#### 2.2.2. Fatty Acids Determination

Fatty acids of citrus beebread are listed in Table 3. Identification and quantification of fatty acids of citrus beebread were determined using GC–MS (Table 3). Fourteen fatty acids were identified in citrus beebread, including eight saturated fatty acids and six unsaturated fatty acids. The ratio of saturated fatty acid in citrus beebread was 39.06% while the unsaturated fatty acid was 60.92%. The major saturated fatty acids were palmitic acid (20.43% ± 0.083%), caproic acid (15.79% ± 0.012%) and stearic acid (1.67% ± 0.004%). Moreover, unsaturated fatty acids, which have been identified in beebread contained one ω-3, two ω-5, one ω-6 and one ω-9 acid. The major polyunsaturated fatty acids (PUFAs) were linoleic acid (35.15% ± 0.144%) (ω-6), γ-linolenic acid (9.45% ± 0.006%) (ω-6) and α-linolenic acid (6.46% ± 0.009%) (ω-3). These results reflected those of Isidorov et al. [18] who also found that large amounts of unsaturated fatty acids (linoleic and α-linolenic) in beebread samples collected from different areas. This finding is consistent with that of a Čeksterytė et al. [19] who found 31 fatty acids in red clover beebread, and who reported the major unsaturated fatty acids as α-linolenic (C18:3 n3) and linoleic (C18:2 n6) acid. Meanwhile, palmitic acid (C16:0) was the highest saturated fatty acid. Čeksterytė et al. [20] published similar findings to our study; 22 identified fatty acids in spring and summer beebread samples. The main fatty acids were arachidonic acid (16.09% ± 2.38%) and oleic acid (15.22% ± 1.35%) and the content of α-linolenic ranged between 1.10 and 8.71%. Additionally, this study suggested that the beebread had a high ratio from ω-6: ω-3 (6.90:1) and it is suitable for human consumption than other plant-derived oils. According to Codex Alimentarius Commission, an acceptable ratio of ω-6: ω-3 is between 1:1 and 5:1 [21]. Thus, the balance of ω-6/ω-3 fatty acids is essential for human health. Recently, Western diets have been found to have a ω-6/ω-3 ratio of 15:1 or 20:1 [22]. A very high ω-6/ω-3 ratio facilitates the pathogenesis of many diseases thus a reduced ω-6/ω-3 ratio can prevent these diseases. Additionally, the ω-6/ω-3 ratio 2:1 and 3:1 suppressed inflammation in patients with rheumatoid arthritis, and the ratio of 5:1 had a beneficial effect on asthma [22]. Therefore, the optimum ratio may vary because chronic diseases are multigenic and multifunctional. On the other hand, Kitts et al. [23] reported that a lower ratio of ω-6/ω-3 fatty acids is more desirable for reducing the risk of many diseases. Also, Nation [24] reported that polyunsaturated fatty acids are essential in beebread for bees.

The deficiency of PUFA causes slow body development, lower productivity and deformed wings. Furthermore, unsaturated fatty acids have many beneficial health effects such as antithrombotic and anti-inflammatory [25]. In addition to reducing the levels of triglycerides and cholesterol in the blood, especially linoleic acid, it is the essential PUFA responsible for reducing LDL cholesterol in the blood [26]. Both omega 3 and omega 6 are essential for body functions. Omega-3 fatty acids are responsible for preventing cardiovascular diseases, diabetes and inflammation diseases [27]; while omega 6 acids are beneficial because humans cannot synthesize them in the human body and should be provided from the diet [11].

### 2.3. Bioactive Properties of Beebread

#### 2.3.1. Total Phenolic and Flavonoid Contents

The healthy benefits and antioxidant activity of beebread referred to phenolic compounds. The amounts of phenolic compounds of ethanolic beebread extract were 10.71 ± 0.593 mg GAE/g dry, while the flavonoids content of citrus beebread was 0.62 ± 0.015 mg quercetin/g dry weight. Zuluaga et al. [3] found that the total phenolic and flavonoid contents of beebread from Columbian were from 2.1 to 13.7 mg GAE/g and 3.2 ± 1.0 mg quercetin/g respectively. Similar results were reported by Urcan et al. [28] found the total phenolic content of five samples from Romania and India of beebread varied from 5.67 to 12.83 mg gallic acid/g. In contrast, Markiewicz-Żukowska et al. [29] found a high amount from total phenolic content (32.78–37.15 mg gallic acid/g) in three samples of ethanol extract from beebread. Additionally, Ivanišová et al. [30] showed the highest amount of total phenolic content in five samples of beebread varied from 12.36–25.4 mg. Zuluaga et al. [3] predicted that the composition of beebread varies widely, being a fermented mixture of bees collected from flora pollen. The main variable in beebread is the pollen composition of the species, which may be influenced by variation in season and area.

#### 2.3.2. HPLC Analysis of Phenolic Compounds

Analysis of the citrus beebread extracted by HPLC showed a complex mixture of phenolic and flavonoids compounds. Results in Table 4 present the concentration and identification of sixteen phenolic compounds of citrus beebread extracted by ethanol 80%. Quercetin and rutin are the major flavonoids compounds, kaempferol 34.02 µg/g is a minor flavonoid compound. Meanwhile, benzoic acid (852.20 µg/g), cinnamic acid (313.80 µg/g) and P-hydroxy benzoic acid (253.6 µg/g), ferulic acid (207.40 µg/g) are the major phenolic compounds in citrus beebread extract. This finding was in agreement with that of Sobral et al. [31] who observed that the main phenolic compounds identified by HPLC were quercetin, kaempferol and myricetin. Meanwhile, Isidorov et al. [18] identified phenolic compounds by GC–MS and showed the major phenolic compounds were (4-hydroxy benzoic, ferulic acid, caffeic acid and p-coumaric) and the major flavonoids were naringin, kaempferol, apigenin and quercetin. The variation of phenolic compound were attributed to the pollen, which can be affected by the season and origin of the pollen based on the flora and the geographical location of flora [13]. Various studies have attributed the effect of beebread as an antioxidant and antitumor activity to the presence of phenolic compounds such as quercetin [32].

### 2.4. Antioxidant Activity

**Figure 1:**
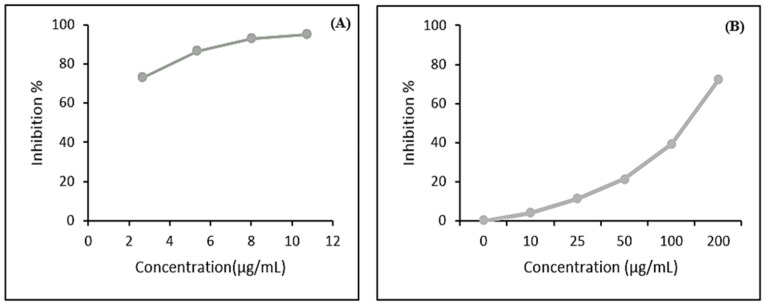
Antioxidant activities of beebread extract (**A**) and BHT (**B**).

The antiradical activity estimated by the DPPH methods is shown in Figure 1A,B. The beebread extract exhibited potential antiradical activity with IC50 of 10.7 µg/mL meanwhile BHT was 39.5 µg/mL. A low IC50 value indicates a high antiradical activity. The antioxidant activity increases with increasing the concentration of beebread extract. These results are similar to Akhir et al. [33], who found that the antioxidant of ethanolic extract of beebread was higher than the antioxidant of propolis estimated by DPPH. Our results agree with Pratap-Singh et al. [34], who reported that antioxidants help favorably regulate the kinetics of lipid peroxidation and polyunsaturated fatty acids degradation.

### 2.5. Antimicrobial Activity of Beebread

**Figure 2:**
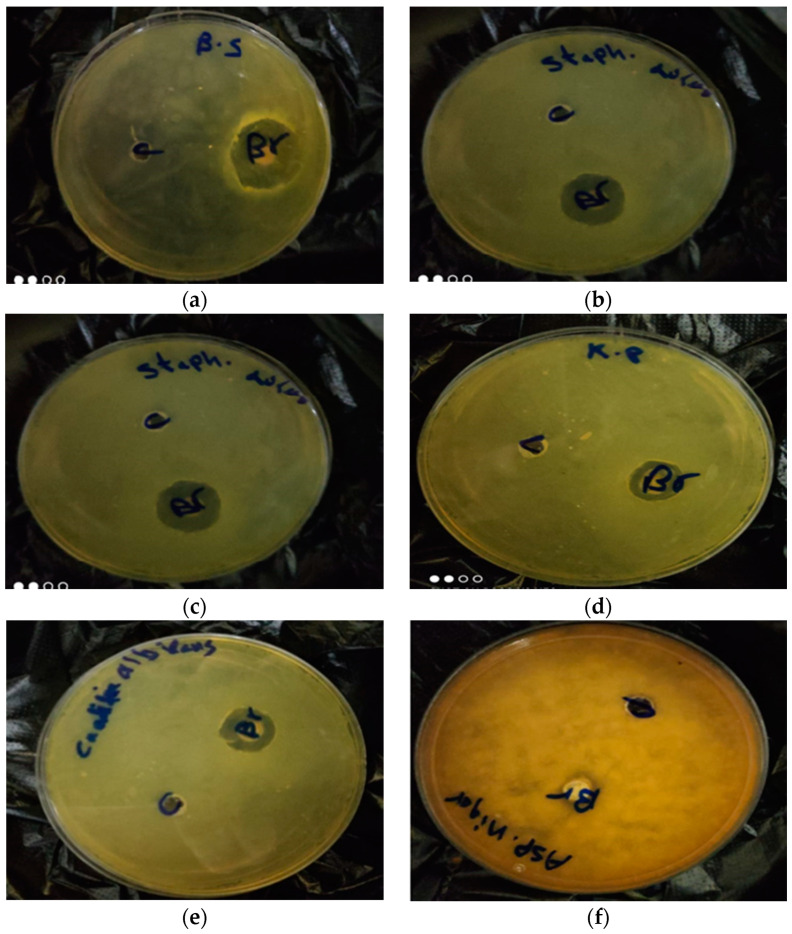
Antimicrobial activities of beebread sample against *Bacillus subtilis* (**a**), *Staphylococcus aureus* (**b**), *Escherichia coli* (**c**), *K. pneumonia* (**d**), *Candida albicans* (**e**) and *Aspergillus niger* (**f**) (inside each plate C for negative control (ethanol 80%) and Br for the bee bread sample).

In the current study, a beebread sample was tested for antimicrobial activity. Table 5 and Figure 2 show the inhibition zone diameter (mm) of the beebread extract against different pathogenic organisms. The results showed that the beebread exhibited high inhibitory activity than the reference compound gentamicin against *Staphylococcus aureus* (26 mm) and *Escherichia coli* (18 mm). Additionally, high inhibitory activity was observed against *Bacillus subtilis* (24 mm), but still less than the reference compound. Similarly, a moderate inhibitory activity of beebread was observed against *K. pneumoniae* (12 mm) and *Candida albicans* (15 mm). While no inhibitory activity was observed against *Aspergillus niger*. As reported previously, beebread’s had potential therapeutic benefits, with strong bacterial activity. Moreover, the Gram-positive bacteria were more sensitive to beebread than Gram-negative bacteria, which may be related to the impermeable outer layer membrane in the Gram-negative bacteria [35,36]. Additionally, according to previous studies beebread had an antimicrobial effect on *C. albicans*, while the activity against fungi varies from moderate inhibitory to no activity [36,37,38].

The antibacterial effect of the beebread extract remains on the flavonoid compounds such as quercetin, kaempferol and rutin. These compounds destroy the bacterial cell by breaking down the cell wall integrity; stop the exchange of ion channels and inhibit the synthesis of adenosine triphosphate (ATP) [12,39]. Additionally, Manning [40] reported that fatty acids such as linoleic, linoleic, lauric and myristic have exerted bactericidal and antifungal properties. This study was in agreement with the results of fatty acids analysis of beebread extract in which different saturated and unsaturated fatty acids were detected. Antimicrobial activity of fatty acids are attributed to impair nutrient intake and direct lysis of bacterial cells [41].

### 2.6. Cell Viability Assay

**Figure 3:**
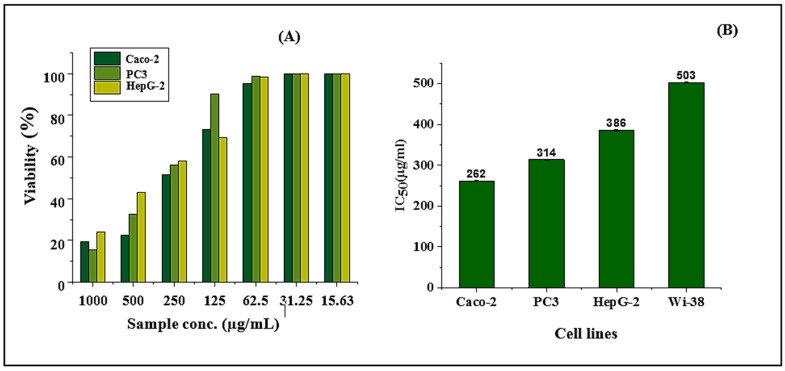
The in vitro anticancer activity of beebread in response to different tumor cell lines as measured with MTT assay (**A**); the IC50 values of beebread against different tumor cell lines (**B**) and (Caco-2: human colorectal adenocarcinoma cell line, human prostate adenocarcinoma cell line; HepG-2: human liver hepatocellular cell line; WI-38: normal lung cell line).

Cancer cell lines are considered the most effective model for the study of the anticancer activity of the different compounds [42]. According to this report the in vitro cytotoxic activity of beebread on the growth of three tumor and normal cell lines; namely Caco-2 (human colorectal adenocarcinoma cell line), (human prostate adenocarcinoma cell line), HepG-2 (human liver hepatocellular cell line) and WI-38 (normal lung cell line) was studied by using the MTT assay. Incubation of carcinoma cells for 24 h in tissue culture medium with different dilutions of samples ranging from 15.63 to 1000 μg mL^−1^, revealed inhibition to be dose-dependent manner (Figure 3A). Beebread has antitumor activity towards Caco-2, PC3 and HepG-2 cell lines with IC50 values 262, 314 and 386 μg mL^−1^, respectively (Figure 3B). While the highest inhibitory effect of beebread was recorded against Caco-2, the viability percent about 19.5%, 22.58% and 51.39% at 1000, 500 and 250 µg mL^−1^, respectively. Followed by PC3 about 15.66%, 32.42% and 55.99%, and HepG-2 about 23.85%, 43.06% and 58.27% at the same concentrations.

While the inhibitory activity of the bee was reduced with decreased concentration of sample from 125 to 15.63 µg/mL. The non or low cytotoxic activity against normal cells is an important consideration when developing material for safe use in a biomedical application. Thus, cytotoxicity assay is considering the first step toward ensuring the biocompatibility of a drug. The present study detected a low cytotoxic effect of beebread against normal cell line with IC50 value 503 μg/mL, which is still higher than IC50 values against three cancer cell lines (Figure 3B).

The different cytotoxicity among different beebread samples remains to the different concentrations of flavonoids and the presence of other compounds [29]. Flavonoids have anticancer activity with a different mechanism of action, among these enhancements of tumor necrosis factors, apoptosis and suppression of cell proliferation [43,44]. The previous studies are consistent with the results of the finding, which concluded that different flavonoid compounds with different concentrations were observed from the HPLC analysis. The quercetin, rutin, cinnamic acid and ferulic acid, respectively the major compounds in the beebread sample with concentrations 717.40, 535, 313.80 and 207.40 μg/g, respectively. Additionally, from GC–MS analysis, linoleic acid was the major compound in beebread with 35.15% peak area followed by oleic acid with 20.43% peak area. Several studies have also reported a suppression in cell proliferation induced by oleic acid and ∝-linolenic acid in different tumor cell lines among these prostate carcinoma cells [31,45].

## 3. Materials and Methods

### 3.1. Collection of Beebread Sample

Beebread samples were collected from honeybee colonies of the local Carniolan hybrid, *Apis mellifera carnica* L. located in Nubaria, Behaira governorate, Egypt during the citrus bloom. Empty combs were added to the colonies to stimulate pollen storage and transform it into beebread. After processing beebread, it was collected manually from combs using a spatula and forceps [44]. Beebread samples were examined to confirm the plant origin, while the rest was kept at −20 °C until laboratory analysis.

### 3.2. Palynological Examination of the Beebread

Beebread sample (10 g) was dissolved with 20 mL of distilled water. The mixture was centrifuged at 1.000× *g* for 10 min and the supernatant was discarded. The residue was redissolved in 20 mL of distilled water and centrifuged again at 1000× *g* for 5 min. The remaining water in the sediment was removed by putting it on an absorbance paper. The residue was distributed on an area of about 20 mm of the slide. The slide was dried up using a hot plate at 40 °C. The pollen grain shape was examined under a light microscope and identified. The frequency of pollen grains was expressed as a percentage of the total pollen amount [46].

### 3.3. Chemicals

All chemicals for analyses were obtained from Sigma Chemical Co., Ltd. (St. Louis, MO, USA).

### 3.4. Preparation of Beebread Extract

Beebread was oven-dried at 45 °C then powdered and filtered at 50 mesh. About 100 g beebread was soaked in 200 mL of ethanol 80% for 24 h and then using a homogenizer for 30 min. The mixture was transferred via filter paper (Whatman No. 1) then the filtrate was concentrated by a rotary evaporator (EYELA, SB-1000, Rikakikai Co. Ltd., Tokyo, Japan) at 40 °C. The concentrate was lyophilized and stored in a tightly closed brown bottle at 5 °C until analysis.

### 3.5. Analysis of Nutritional Value of Beebread

The macronutrient analysis, including total lipids, ash and protein were determined by standard methodology described by AOAC [47]. Total carbohydrates were estimated by difference and the energy was estimated as per the following equation [48]

Energy (kcal) = 4 × (g protein + g carbohydrates) + 9 × (g fat)

### 3.6. Mineral Composition

The minerals content of beebread sample was examined using inductively coupled plasma-atomic emission spectroscopy (ICP-OES: Icap6000 serious, Serial No. Icp-20080614, Thermo Scientific,. Cambridge, UK). Each sample was measured twice to determine the macroelements such as Na, K, Ca, Mg and P, and the microelements such as Zn, Mn and Fe [49].

### 3.7. Free Sugars

Sugar composition (glucose, fructose and sucrose) was analyzed by Agilent Technologies 1100 series liquid chromatography with an autosampler and a refractive index detector. The analytical column was SCR-101N. The mobile phase was water and the flow rate was 0.7 mL/ minute. The optimized temperature of the oven was 40 °C.

### 3.8. Identified Fatty Acids Using Gas Chromatography–Mass Spectrometry Analysis (GC–MS)

The GC–MS system (Agilent Technologies) was equipped with gas chromatograph (7890B) and mass spectrometer detector (5977A) at Central Laboratories Network, National Research Centre, Cairo, Egypt. The GC was equipped with DB-WAX column (30 m × 250 μm internal diameter and 0.25 μm film thickness). Analyses were carried out using hydrogen as the carrier gas at a flow rate of 1.9 mL/min at a splitless, injection volume of 1 µL and the following temperature program: 50 °C for 1 min; rising at 25 °C /min to 200 °C and held for 5 min; rising at 3 °C/min to 220 °C and held for 10 min and rising at 5 °C/min to 240 °C and held for 8 min. The injector and detector were held at 250 °C and 290 °C, respectively. Mass spectra were obtained by electron ionization (EI) at 70 eV and using a spectral range of m/z 60–400 and solvent delay 6 min. Identification of different constituents was determined by comparing the spectrum fragmentation pattern with those stored in Wiley and NIST Mass Spectral Library data.

### 3.9. Bioactive Properties of Beebread

Total phenolic content of beebread sample was determined as protocol described by Wiktor et al. [50] using the Folin–Ciocalteu system with minor changes. In general, 50 µL of each sample (pre-evaporation extract) was mixed with 250 µL distilled water and 50 µL of Folin–Ciocalteu reagent and vortexed. After 3 min incubation at room temperature, 150 µL of sodium carbonate (7.5%) was added. The mixture remained in the dark for 1 h and the absorbance at 750 nm was measured. The total content of phenolics was determined using a standard gallic acid curve and expressed as a sample of mg gallic acid/mg.

Total flavonoids content beebread sample was measured by a colorimetric assay developed by Abedelmaksoud et al. [51,52], with modifications. Briefly, 250 µL of extract or standard solution of catechin at different concentration (20–260 µg/mL) and 1 mL of distilled water were mixed in a 10 mL test tube. The following were successively added: at zero time, 75 µL of 5% NaNO_2_; at 5 min, 75 µL of 10% AlCl_3_ and at 6 min and 500 µL of 1 N NaOH. The solution was then immediately diluted by adding 2.5 mL of distilled water and mixed thoroughly. A microplate reader directly measured the absorbance of the mixture (pink color) at 510 nm against a blank sample and the results were expressed as catechin equivalents (mg CE/g).

HPLC analysis of phenolic compounds in the beebread sample was conducted as follows the beebread extract was analyzed using an Agilent 1260 series HPLC system (Agilent technologies Inc., Santa Clara, CA, USA). The separation was passed using C18 column (100 mm × 4.6 mm i.d., 5 μm). The mobile phase contained of (A) water 0.2% H_3_PO_4_, (B) methanol and (C) acetonitrile at a flow rate 0.6 mL/min. Gradient elute was as per the next scheme: 0–11 min (96% A, 2% B); 11–13 min (50% A, 25% B); 13–17 min (40% A, 30% B); 17–20.5 min (50% B, 50% C) and 20.5–30 min (96% A, 2% B). Detection wavelength (UV detector) was set at 284 nm. The injection size was 20 μL and the column temperature was maintained at 30 °C. Compounds were known by comparing their retention time with those from authentic standards. Calibration curves were utilized to evaluate the compound amounts.

The antioxidant activity of each sample was measured according to the Altemimi et al. [53] method of DPPH (2, 2-diphenyl-1-picrylhydrazyl) and estimated according to the next equation: (Antioxidant activity% = [(absblank − abssample)/absblank] ∗ 100). where, absblank and abssample refer to the absorbance of blank and sample respectively. The necessary concentration of the sample to inhibit 50% of the free radicals (IC50) was calculated.

### 3.10. Antimicrobial Activities

The antimicrobial activities of the beebread sample were evaluated by the agar diffusion technique against Gram-negative *Escherichia coli* (ATCC 8739) and *K. pneumonia* (ATCC 13883) also, Gram-positive *Staphylococcus aureus* (ATCC 6538) and *Bacillus Subtilis* (ATCC 6633), unicellular fungi *Candida albicans* (ATCC 10221) and filamentous fungi (*Asp. niger*). Amphotericin B and gentamicin (10 mg/mL) were used as the standard antimicrobial agent. Antimicrobial activity was expressed as the diameter of inhibition zones (mm) [54].

### 3.11. The Viability of Control and Treated Cells Were Evaluated Using the MTT Assay

Briefly, Caco-2 (human colorectal adenocarcinoma cell line), PC3 (human prostate adenocarcinoma cell line), HepG-2 (human liver hepatocellular cell line) and WI-38 (normal lung cell line) were seeded in 96-well plates containing 100 µL of the growth medium at a density of 1 × 104 cells/well. Cells were permitted to adhere for 24 h till confluence, washed with PBS, and then treated with different concentrations of compounds in a fresh maintenance medium from 1000 to 15.63 µg mL^−1^ and incubated at 37 °C for 24 h. After treatment (24 h) the cells in each well were incubated at 37 °C with 100 µL of MTT solution (5 mg/mL) for 4 h. After that, the MTT solution was removed, and then 100 µL of DMSO was added to each well. The absorbance was read at 570 nm using a microplate reader (SunRise TECAN, Inc., San Jose, CA, USA) [55].

### 3.12. Statistical Analysis

Triplicate chemical assays were conducted out and the information was expressed as mean ± standard deviations.

## 4. Conclusions

Beebread is a neglected bee product treasure, consisting of pollen mixed with honey and bee digestive enzymes. The chemical composition of beebread is not stable and depends on the surrounding flora characteristics. Despite the high nutritional value, few researchers are concerned about its composition compared to other bee products. This is mainly because of the difficulty of its production. Egyptian beebread is a good source of carbohydrates, protein, minerals, fatty acids and phenolic compounds. Moreover, the phenolic and flavonoids compounds have a major antioxidant, antimicrobial and antitumor effect. Results showed that Egyptian beebread is a suitable nurturant and a natural therapeutic, which has antioxidant, antitumor and antimicrobial activity for humans. In an overview, in the coming future Egyptian beebread can be recommended to fortify baby food (as a good source for protein and omega acids that help prevent cancer).

## Figures and Tables

**Table 1:** Chemical composition (g/100 g BB), energy value (Kcal/100 g BB) and free sugars (%) in beebread.

Chemical Composition		Free Sugars	%
Protein	23.58 ± 0.183	sucrose	1.775 ± 0.017
Ash	2.87 ± 0.108	glucose	7.136 ± 0.452
Lipids	9.45 ± 0.079	Fructose	11.355 ± 0.555
Carbohydrates	64.10 ± 0.391	Total	20.266 ± 0.930
Energy	435.77 ± 2.904		

**Table 2:** The mineral composition of beebread (mg/100 g).

Macro Minerals	(mg/100 g)
K	290.202 ± 2.645
P	215.202 ± 1.103
Ca	170.200 ± 3.995
Mg	60.100 ± 4.369
Fe	31.900 ± 1.153
Na	11.250 ± 0.687
Zn	8.730 ± 0.644
Mn	2.660 ± 0.251

**Table 3:** GC–MS analysis of fatty acids in citrus beebread, (mean ± SD).

Fatty Acid	Relative Area Percentage (%)
Caproic acid (C6:0)	15.79 ± 0.012
Caprylic acid (C8:0)	0.42 ± 0.005
Lauric acid (C12:0)	0.19 ± 0.006
Myristic acid (C14:0)	0.18 ± 0.005
Myristoleic acid (C14:1 n5)	0.21 ± 0.006
Pentadecylic acid (C15:0)	0.09 ± 0.003
Palmitic acid (C16:0)	20.43 ± 0.083
Margaric acid (C17:0)	0.29 ± 0.003
Stearic acid (C18:0)	1.67 ± 0.004
Oleic acid (C18:1n 9)	3.36 ± 0.004
Linoleic acid (C18:2)	35.15 ± 0.144
γ-Linolenic acid (C18:3 n6)	9.45 ± 0.006
α-Linolenic acid (C18:3 n3)	6.46 ± 0.009
Glycidyl oleate (C21:0)	6.29 ± 0.008
SFA%	39.06 ± 0.036
MUFA%	9.86 ± 0.012
PUFA%	51.06 ± 0.090
Unknown	0.02 ± 0.001

**Table 4:** Total phenolic compounds and identification of phenolic compounds.

Compounds	µg/g	RT
Gallic acid	1.60 ± 0.05	3.967
p-Hydroxy benzoic acid	253.64 ± 0.09	7.539
Catechin	11.59 ± 0.03	8.773
Vanillic acid	20.32 ± 0.02	9.758
Caffeic acid	4.198 ± 0.03	10.142
Syringic acid	0.948 ± 0.02	10.496
Benzoic acid	852.20 ± 0.10	14.573
Ferulic acid	207.40 ± 0.08	15.374
Rutin	535.00 ± 0.11	16.682
Ellagic acid	16.34 ± 0.04	16.897
o-Coumaric acid	10.90 ± 0.06	17.528
Resveratrol	53.20 ± 0.07	19.346
Cinnamic acid	313.80 ± 0.09	20.123
Quercetin	717.40 ± 0.17	21.639
Myricetin	24.50 ± 0.06	23.312
Kaempferol	34.02 ± 0.08	24.320

**Table 5:** Antimicrobial activities of beebread sample against different pathogenic microorganisms.

Pathogen	Beebread	Standard
Gram-positive bacteria		Gentamicin
*Staphylococcus aureus* (ATCC 6538) *Bacillus Subtilis* (ATCC 6633)	26 ± 0.81 24 ± 0.53	15 ± 0.74 25 ± 0.48
Gram-negative bacteria		Gentamicin
*K. pneumoniae* (ATCC 13883) *Escherichia coli* (ATCC 8739)	12 ± 0.39 18 ± 0.60	22 ± 0.75 17 ± 0.37
Fungi		Amphotericin B
*Candida albicans* (ATCC 10221) *Aspergillus niger*	15 ± 0.73 NA	21 ± 0.59 15 ± 0.64

## Data Availability

All data is included within the manuscript.

## Sample Availability

Not available.
